# 

**Published:** 1813-03

**Authors:** 


### MONTHLY CATALOGUE OF MEDICAL BOOKS.

A Comprehensive View of the Small-pox, Cow-pox, and Chicken-pox; with a concise History of their different Stages and Terminations. By James Saunders, M.D. 8vo.—Longman and Co.

An Essay on the Absorbents; comprising some Observations upon the relative Pathologies and Functions of the Absorbent and Secreting Systems. By Daniel Pring, Surgeon, Bath. 8vo.—Callow.

An

An Introduction to Medical Literature, including a System of Practical Nosology; intended as a Guide to Students, and an Assistant to Practitioners. Together with detached Essays on the Study of Physic; on Classification; on Chemical Affinities; on Animal Chemistry; on the Blood; and on the Medical Effects of Climates. By Thomas Young, M.D. F.R. and L.S. 8vo.—Underwood and Co.

A Practical Treatise on the Cataract. By John Stevenson. 8vo. —Highley and Co.

A View of the Progress and Present State of Animal Chemistry. By Jöns Jacob Berselius, M.D. Translated from the Swedish. By Gustavus Brunnark, D.D. 8vo.—Hatchard.

History of James Mitchell, a Boy born Blind and Deaf; with an Account of the Operation performed for the Recovery of his Sight. By James Wardrop, F.R.S. Edinb. 4to.—Murray.

Cases of Hydrophobia, including Dr. Schoolbred's and Mr. Tymon's successful Cases; with some Observations on the Nature and Seat of the Disease. By J. O'Donnel, M.D. 8vo.—Callow.

---

### ADDRESS TO THE FACULTY.

*As information variously useful to the Profession and the Public, we purpose to insert, from month to month, a Medical View of the Counties of the United Kingdom, and we invite the communications of all our correspondents to enable us to perfect this design. Our idea in the execution of such a plan, is to give the names and residence of the Physicians, the Surgeons, and Apothecaries in each County, distinguishing in general terms the departments of the profession in which they usually practise when it is of a particular character. We wish also to receive notices of any local Medical Institutions, with the names of the Presidents and Secretaries; and also lists of Hospitals, Infirmaries, &c. &c. with the names of the Medical Assistants, and of the resident Surgeon or Apothecary, and Secretary. We design to introduce one or two counties per month, and, as we shall prefer an alphabetical arrangement of the counties as nearly as may be, we invite the immediate transmission of lists from* ANGLESEA, BERKSHIRE, BEDFORDSHIRE, BUCKINGHAMSHIRE, BAMPSHIRE, CHESHIRE, CUMBERLAND, CARMARTHENSHIRE, CARNARVONSHIRE, &c.

---

### *NOTICES TO CORRESPONDENTS.*

*The Drawing for Mr. Shillito's Case, we have not yet been able to get from the artist employed on it; but we have no doubt of being able to present it to the public in our next Number.*

*Want of room has obliged us to omit our usual article of Collectanea, as well as several original papers; amongst others we have to acknowledge communications from Dr. Clarke; Messrs. Smith; Cornish; Brown; and Weatherhead; J. W.; M. N.; Salus Publica; An Apothecary; &c. &c. &c.*

